# Monthly Summary

**Published:** 1874-02

**Authors:** 


### MONTHLY SUMMARY.

*Incombustible Paper and Fire-proof Ink.*—An invention has been recently patented in this country, which, if it will only stand the test, should have a wide field of usefulness. A really incombustible paper, without a fire-proof ink, would be a very valuable article in many businesses, and for many purposes of every-day life, but if it can be supplemented by a fire-proof ink, its value will be enhanced tenfold. Such a discovery Mr. G. W. Halfpenny believes he has made, and has accordingly securd his rights by obtaining the Great Seal. We gather from his specifications that the paper prepared by his process is absolutely indestructible by fire of any degree of fierceness, but that under such circumstancees as fires in houses, factories, or other buildings, it is "ordinarily incombustible." The inventor prepares his paper in the usual manner, from a pulp consisting of vegetable fiber, asbestos, alum and borax, in about the following proportions: Vegetable fiber, one part; asbestos, two parts; borax, one-tenth part; and alum, two-tenths of a part. The vegetable fibers are minutely divided, and treated in the manner usual in the production of ordinary paper: the asbestos is also divided as much as possible, and the two are theu intimately mixed with the alum and borax in a sufficient quantity of water to make a pulp of the requisite consistency, which is then made into paper by any of the well-known processes. The proporitions given are not rigid, but may be varied to suit the quality and nature of the desired product, and also to suit the different qualities of the raw materials.

Thus, the inventor says he has made incombustible paper in which the proportions of the ingredients varied from 50 to 70 parts of asbestos, and from 30 to 50 parts of flax or other vegetable fiber, with only two and a half per cent. each of alum and borax. He proposes to use in some cases silicate of soda, in order to insure hardnesss and coherence in the substance of the paper after it has been acted upon by fire. In order, we presume, to obtain a paper of great strength and flexibility, the sheets may be made of linen or other woven fabric, and coated on both sides with the incombustible paper.

The fire-proof ink for use in writing or printiug on the incombustible paper is made of the following substances in or about the proportions given, in apothecaries' weight: Graphite, twenty-two drachms; copal or other rosinous gum, twelve grains; sulphate of iron, two drachms; tincture of nut galls, two drachms; and sulphate of indigo, eight drachms. These materials are mixed together and boiled in water, the graphite, of course, being reduced to an impalpable powder. This ink, which besides being fire-proof is said to be insoluble in water, under ordinary circumstances is black; but when colored inks are desired, the graphite is replaced by an earthy or mineral pigment of the desired color.

Another portion of this invention consists in the application of the incombustible paper pulp to the manufacture of covers for books, and of wrappers or envelopes for parcels and packages. The inventor also utilizes talc for this purpose, singly or in combination, with his incombustible mill-board or ordinary boards. Thus the "back" of the book may consist of a number of slips of talc over lapping one another, secured to and supported by a piece of incombustible paper.—*English Mechanic.*

---

*The Use of Sewing Machines.*—During the late session of the State Medical Society of Virginia, at Staunton, Dr. Parker read a very interesting paper on the subject of sewing machines and their effect on the health of women, in which the conclusions were announced:

First. That fatigue is not disease, and that there is no reason to conclude that the use of the muscles employed in machine-work for a reasonable time is injurious. Second. That the machine may be used for four or five hours daily in a family, by a lady in ordinary health, without injury. Third. That the damage to health in the factory is due to the hygienic conditions under which the work is done, and the natural delicacy of some of the operatives, unfitting them for long-continued labor of any kind. Fourth. That the sewing-machine is a great boon to womenkind, increasing her compensation, protecting her sight, and in the family lessening her labors.—*Med. and Surg. Reporter.*

---

*Counterfeit Diplomas.*—The establishment on Pine street, Philadelphia, where medical degrees, regular and honorary, are conferred at so much a head, has been several times sharply overhauled, but has continued prosperous in spite of its overhauling. The greater the discredit into which the "American University of Philadelphia" has fallen at home, the greater the energy with which its business has been pushed at a distance, and the more lucrative its returns have been.

It must be allowed that there is a large element in human nature that likes to be humbugged ; otherwise such frail securities as El Paso railroad bonds and Emma mining stocks would never have had a successful run in foreign exchanges, where nothing authentic was known about them, and from the nature of the case it was impossible that anything could be known. Upon this same credulity and conceit, manifested in a different way, the shrewd Yankee operators in university titles have played successfully, and they have found the market lucrative enough to pay for great risks at home. There is a good prospect now that this particular kind of swindling will be brought to an end. Responsible charges have at last been filed, and the "American University" will be called to answer upon what warrant its proceedings are based. The charges come this time from Germany, where the sale of bogus titles has been vigorously carried on through resident agents. The chief agency was in charge of "Dr. P. F. A. Vander Vyver, LL.D.," Jersey, England, to whom all persons who wished to be promoted to any degree by this university, without being personally present, were to send their applications, and $120 in money—being the total cost of the diploma.

This eminent doctor of laws advertised extensively in German and other European papers, setting forth the facility of getting degrees in this way, without the usual inconvenience of previous study, or even of a Journey to America and back again. Here and there gentlemen of moderately good standing among scholars, have been tempted by Dr. Vander Vyver's prospectuses to bid for these paper titles, and have worn them with great complacency, till the swindle was fully explained to them. The authorities of Philadelphia, in company with the officers of the University of Pennsylvania, with which the bogus university has been confounded, are not at work. with the evidence of its deception in their hands, to bring its officers to justice, or, failing in that, at least to put a stop to their business.—*Boston Daily Advertiser.*—*Boston Med. and Surg. Journal.*

---

*Bones from Dissecting-Rooms.*—An extraordinary quantity of human bones, says the *Lond. Med. Times*, has been found in the excavation for the foundation, upon which workmen have been for some time engaged, of the new wing of the London Hospital, the site of which will be the old Medical College. A shed which stands in a corner adjoining the works is filled to the roof with these bones. Besides these, no less than fifty-two coffins, filled with bones, have been removed to Ilford for re-interment. It is calculated that what remains will fill thirty more coffins. The contents of each coffin weigh two hundred-weights, making in all about eight tons of bones. Taking the average weight of a human skeleton to be fourteen ponnds, it is found that this shed contains all that now remains of no less than 1,280 human beings. They are in an excellent state of preservation, though they have evidently been beneath the ground for a great number of years. It may be mentioned that at the time the great Windmill-street School of Anatomy ceased to exist, an immense quantity of bones was found in deep, dry wells, the accumulation from the dissecting-room of probably half a century.—*Druggists' Circular.*

---

*Children Versus Crime.*—Bertillon, in some interesting statistics relating to the population of France, laid before the Academie de Medecine not long since, stated that in a million married persons without children there is an average of 175 convicts yearly; while in the same number with chlldren, there are but 109. In a million childless married men, 470 commit suicide annually, while in an equal number with children, only 205 make way with themselves; among married women the proportion was 157 to 45 ; widowers with children, 526 ; without children, 1004 ; widows with children, 104 ; without, 238.—*Med. and Surg. Reporter*

---

*The Legal Value of Teeth.*—A curious law suit was recently tried in a New Hampshire court. A widower brought suit for damages to the amount of $5,000 against a dentist who etherized his late wife and extracted more teeth than she meant to part with. It seemed to have been a blunder on the part of the dentist, and the jury, apparently being in doubt how far a husband suffered such injury from the loss of a jaw power on the part of the wife as could be compensated in damages, brought in a nominal verdict for the plaintiff.—*Med. and Surg. Reporter.*

